# Ultrasound-Guided Nerve Blocks for Patients with Clavicle Fracture in the Emergency Department

**DOI:** 10.3390/jcm15020523

**Published:** 2026-01-08

**Authors:** Cheng-Chien Chen, En-Hsien Su, Hua Li, Kar Mun Cheong, Yung-Yi Cheng, Su Weng Chau, Yi-Kung Lee, Tou-Yuan Tsai

**Affiliations:** 1 Department of Medicine, National Taiwan University Hospital, Taipei City 100225, Taiwan; 108311128@gms.tcu.edu.tw; 2 School of Medicine, Tzu Chi University, Hualien 97004, Taiwan; 108311106@gms.tcu.edu.tw (E.-H.S.); dm015377@tzuchi.com.tw (S.W.C.); a122514@tzuchi.com.tw (Y.-K.L.); 3 Department of Medicine, Taipei Tzu Chi Hospital, Buddhist Tzu Chi Medical Foundation, New Taipei City 231016, Taiwan; 4 Emergency Department, Dalin Tzu Chi Hospital, Buddhist Tzu Chi Medical Foundation, Chiayi 622401, Taiwan; dl43322@tzuchi.com.tw (H.L.); dl27404@tzuchi.com.tw (K.M.C.); 100311024@gms.tcu.edu.tw (Y.-Y.C.); 5 Center for Health Data & Advanced Translational Analytics, Dalin Tzu Chi Hospital, Buddhist Tzu Chi Medical Foundation, Chiayi 622401, Taiwan; 6 School of Post-Baccalaureate Chinese Medicine, Tzu Chi University, Hualien 97004, Taiwan; 7 Institute of Epidemiology and Preventive Medicine, College of Public Health, National Taiwan University, Taipei 100025, Taiwan

**Keywords:** clavicle fractures, pain management, acute pain, nerve block, regional anesthesia, ultrasound-guided procedures, emergency service, opioid analgesics

## Abstract

**Background**: Opioids and nonsteroidal anti-inflammatory drugs (NSAIDs) for clavicle fracture pain management carry significant adverse effect and allergic reaction risks. This study assessed ultrasound-guided nerve block (USNB) efficacy for acute clavicle fracture pain in emergency department (ED) patients, providing an alternative to NSAIDs and opioids with fewer adverse effects. **Methods**: This retrospective, single-center observational study was conducted in accordance with Methods of Medical Record Review Studies in Emergency Medicine Research guidelines. Adult patients (≥20 years) who presented to the ED with traumatic clavicle fractures between 1 January 2015 and 30 November 2023 were included. Of the 343 eligible patients, 12 received ultrasound-guided nerve blocks (USNB) and 331 received standard care. To improve exchangeability, 1:10 matching with replacement was performed according to patients’ characteristics, such as age, sex, initial pain score, and comorbidities. The primary outcome was pain relief, assessed via the pain intensity difference (PID) on the Numerical Rating Scale within 360 min post-intervention. Meaningful pain relief was defined as a PID ≥ 4. Secondary outcomes included rescue opioid use, ED length of stay, hospital length of stay, and USNB-associated complications, such as vascular puncture, nerve injury, or local anesthetic systemic toxicity. Data were analyzed using time-course, time-to-event (time to meaningful pain relief), and linear regression analyses. **Results**: A total of 12 patients in the USNB group and 85 matched patients in the standard care group were analyzed after baseline characteristics matching with replacement. Compared to standard care, USNB was associated with significantly greater pain relief (*p* < 0.001). In the time-to-event analysis, USNB led to a 3.41-fold faster achievement of meaningful pain relief compared with that achieved with standard care (HR = 3.41; 95% CI, 1.47–7.90; *p* = 0.004). No significant differences were observed between groups in rescue opioid use, ED length of stay, or hospital length of stay. No USNB-associated complication developed in the USNB group. **Conclusions**: In patients with traumatic clavicle fractures, USNB provides more rapid and sustained pain relief than standard analgesic care in the ED, without increasing the ED length of stay. Large prospective studies are needed to confirm these findings.

## 1. Background

Clavicle fractures are common skeletal injuries, accounting for 2.6–4% of all fractures and 44% of those in the shoulder girdle [1,2]. As one of the most frequently encountered upper extremity injuries, they represent a significant proportion of emergency department (ED) presentations [3]. These injuries frequently result from falls onto the shoulder or motor vehicle accidents, with the highest incidence occurring among young adult males [2,4,5]. These fractures can cause significant pain, necessitating effective acute pain management in the ED [2].

The standard approach to pain management for patients with clavicle fractures involves a multimodal strategy including acetaminophen, nonsteroidal anti-inflammatory drugs (NSAIDs), and opioids [2]. However, this approach has limitations; NSAIDs carry risks of gastrointestinal bleeding and renal impairment [6,7], whereas opioids are associated with nausea, sedation, cognitive impairment, and respiratory depression; furthermore, opioids present significant potential for abuse and misuse [7,8]. The ongoing opioid epidemic highlights the need for effective alternatives to reduce opioid exposure and its associated harms [8,9].

Regional anesthesia (RA) has emerged as an effective alternative to traditional pain relief in the ED, offering targeted, long-lasting analgesia with fewer systemic side effects [10,11,12]. The clavicle exhibits complex sensory innervation, receiving contributions from the superficial cervical plexus (anterior rami of C1–C4), the brachial plexus (anterior rami of C5–C7), and other cervical spinal root branches [10,11,13]. The supraclavicular, subclavian, long thoracic, and suprascapular nerves are implicated [10,13]. Consequently, several RA techniques have been explored for clavicle fractures, including the superficial cervical plexus block, interscalene block, and clavipectoral plane block, often administered under ultrasound guidance for precision and safety [10,11,13].

Identifying alternative analgesic strategies is essential for transitioning away from an overreliance on opioids [7,8,9,11]. Ultrasound-guided nerve blocks (USNBs) offer a promising solution, providing site-specific pain control that can significantly reduce opioid consumption and associated adverse effects [7,14,15,16]. In geriatric patients with hip fractures, for example, ultrasound-guided femoral nerve block has been shown to reduce delirium, decrease the hospital length of stay, and improve functional outcomes by providing effective pain relief with minimal systemic side effects [7,15,16,17]. Similarly, for clavicle fractures, USNBs such as the superficial cervical plexus block have demonstrated effectiveness in providing rapid and significant pain relief, potentially reducing opioid dependence and improving overall patient outcomes [11,18]. The use of ultrasound guidance enhances the safety and success rate of these procedures compared with those achieved with traditional landmark-based techniques [7,11,14,16].

Despite the established benefits of RA in various settings, its application for clavicle fractures in the ED remains understudied [11,13,19]. Most existing studies are limited by small sample sizes or short-term pain assessments; thus, robust evidence supporting the long-term efficacy and safety of USNBs for this indication is lacking [4,7,10,11].

This study aimed to evaluate the effectiveness of single-shot USNBs in reducing acute pain among patients with clavicle fractures presenting to the ED by using a retrospective single-center electronic medical record review.

## 2. Materials and Methods

### 2.1. Study Design and Protocol

This retrospective, single-center observational study was conducted at a high-volume ED within an academic medical center with approximately 41,000 annual patient visits. The study received ethical approval from the Institutional Review Board of Dalin Tzu Chi Hospital, Buddhist Tzu Chi Medical Foundation, Taiwan (approval number B11304032). Given the retrospective nature of this study and absence of direct patient contact, the requirement for informed consent was waived by the institutional review board. The study design adhered to recommendations detailed in the “Methods of Medical Record Review Studies in Emergency Medicine Research” [20].

### 2.2. Study Population and Setting

Adult patients who presented to the ED with a traumatic clavicle fracture between 1 January 2015, and 30 November 2023, were included. The diagnosis was confirmed using International Classification of Diseases (ICD) codes from ED visit records. Patients were excluded if they were younger than 20, transferred from an outpatient department, refused consent, had incomplete follow-up data, had known allergies to opioids or local anesthetics, presented with multiple fractures, or were classified as triage level 1 on arrival. Data were collected by two investigators who were blinded to the hypothesis, with interobserver reliability assessed.

### 2.3. Interventions

USNBs were performed by emergency physicians (EPs) certified by a regional anesthesia training board when available. In a retrospective manner, the patients were divided into two cohort groups: the USNB group and the standard care group, and patient data were retracted from the electronic health record system. In the USNB group, clinicians selected the nerve block technique based on personal preference, such as interscalene nerve block and superficial cervical plexus block. The procedural standards for related blocks are summarized as follows. Interscalene block involves placing a high-frequency linear probe over the interscalene groove at the level of the cricoid cartilage to visualize the C5–C7 nerve roots. After identifying the characteristic “traffic-light” arrangement of the roots, a needle is advanced in-plane toward the plexus, and local anesthetic is injected to surround the nerve roots. This technique is particularly effective for shoulder and proximal humeral analgesia [21]. Superficial cervical plexus block is performed with the transducer placed posterior to the sternocleidomastoid (SCM) muscle at the midpoint of its length. The superficial cervical plexus is visualized lateral to the SCM fascia. An in-plane needle approach is used to deliver the anesthetic into the superficial fascial plane, allowing diffusion across the sensory branches (e.g., lesser occipital, great auricular, transverse cervical, and supraclavicular nerves) [22]. EPs were permitted to select the nerve block technique based on their individual expertise, choosing the method they were most proficient with or combining techniques when clinically appropriate to optimize analgesic effect. A total of 10 mL of 1% lidocaine was injected adjacent to the target. Lidocaine was chosen for its rapid onset, which is ideal for the ED setting [7,23]. In the standard care group, patients were treated with parenteral or oral analgesics—such as opioids, NSAIDs, and acetaminophen—selected at the discretion of the EPs and guided by the World Health Organization analgesic ladder. The goal was to achieve a 50% reduction in pain or to adjust treatment based on patient request.

### 2.4. Measurements

Pain intensity was assessed using the 11-point NRS, a validated and widely used patient-reported outcome measure with strong psychometric properties, where 0 indicates no pain and 10 indicates the worst imaginable pain, at baseline and at multiple intervals up to 360 min post-intervention. The primary outcome was pain relief, evaluated using the pain in difference (PID) and calculated as the difference between the baseline NRS score and the score at each subsequent time point. Pain intensity was measured using the 11-point NRS at predetermined intervals—baseline (0 min), 15 min, 30 min, and 1, 2, 3, and 4 h post-intervention—to capture both the early onset and sustained analgesic effects of the treatments. Use of PID as a primary outcome is well-established in both chronic and acute pain research, and has been validated as a clinically meaningful measure of pain reduction [24]. Meaningful pain relief was defined as a PID ≥ 4, a threshold previously established as a clinically significant indicator of pain reduction with high specificity [25]. The time to meaningful pain relief was recorded as the time from intervention completion to the first report of a PID ≥ 4. Total opioid exposure was standardized and reported in morphine milligram equivalents (MMEs), an equianalgesic conversion metric recommended in opioid-related clinical research to enable comparison across agents with differing potencies.

### 2.5. Outcomes

The primary outcome measure was the (PID) between the USNB and standard care groups. Secondary outcomes included opioid consumption (MME), ED length of stay, hospital length of stay, and the occurrence of complications, including nerve injuries, vessel injection, hematoma, local anesthetic systemic toxicity, allergic reactions, pneumothorax, hemidiaphragmatic paresis and any other adverse events considered attributable to the intervention.

### 2.6. Statistical Analyses

Patient characteristics were summarized using means, standard deviations (SDs), and percentages. For categorical variables, the chi-square test was used for statistical comparisons. When any expected cell frequency was less than 5, Fisher’s exact test was employed to ensure accurate estimation. To enhance exchangeability between USNB and standard care groups, we employed k-nearest-neighbor matching. Using a 1:10 matching ratio with replacement, patients in standard care group were matched to patients in USNB group based on age, sex, initial pain score, and comorbidities to construct balanced comparison cohorts. The matching algorithm proceeded sequentially: First, k-nearest-neighbor matching (k = 10) was performed for each USNB patient. Second, exact matching on sex was required. Third, distance metric matching was applied for age, initial pain score, and comorbidities using Manhattan distance (L1 norm) with relative calipers. Matching was performed with replacement among control candidates satisfying caliper restrictions.

For primary outcome, the time-course analysis of PID was performed using a continuous autoregressive model with restricted cubic splines to model nonlinear relationships over time and using likelihood ratio test to test the significance of trajectories between groups. Time-to-event analysis for meaningful pain relief was conducted using Kaplan–Meier plots with a log-rank test and a Cox proportional hazards regression model to estimate hazard ratios (HRs) and 95% confidence intervals (CIs). Right censoring of time-to-event data occurred when: (1) patients did not achieve meaningful pain relief (PID ≥ 4) by observation completion, (2) patients withdrew prior to the event, or (3) rescue analgesia was administered. Censored observations indicate the event had not occurred by the censoring time, with the true event time (if it occurred) remaining unobserved. For example, if a patient withdrew after 6 h without meaningful pain relief, their observation was censored at 6 h. For secondary outcome, linear regression analysis was performed to analyze the outcome measures, including MME, duration of ED stay, and duration of hospital stay. All analyses were performed using R Statistical Software version 4.4.1. A *p* value < 0.05 was considered to indicate significance.

## 3. Results

### 3.1. Characteristics of the Study Participants

**Figure 1:**
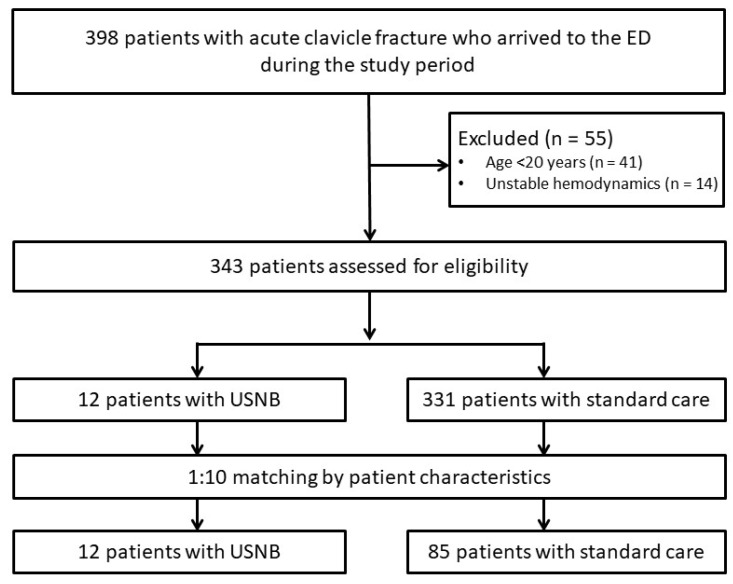
Flow diagram of the selection of study subjects. USNB: ultrasound-guided nerve block.

During the study period, 398 patients with acute clavicle fractures presented to the emergency department. A total of 55 patients were excluded, including 41 patients younger than 20 years and 14 patients with unstable hemodynamics, leaving 343 patients eligible for initial assessment (Figure 1). Among these, 12 patients received USNB, while 331 patients received standard care. To reduce baseline imbalance and enhance comparability, a 1:10 matching procedure with replacement was applied based on predefined patient characteristics. After matching, 97 patients were included in the final analytic cohort, comprising 12 patients in the USNB group and 85 matched patients in the standard care group. The patients’ baseline characteristics are summarized in Table 1. The mean age was comparable between groups (60.9 ± 14.7 years in the USNB group vs. 59.9 ± 13.9 years in the standard care group, *p* = 0.8). The proportion of women was similar (50.0% vs. 45.9%, *p* = 0.8), as were the baseline BMI values (22.7 ± 3.4 vs. 23.9 ± 4.6 kg/m^2^, *p* = 0.4). Initial pain severity on presentation did not differ significantly, with a mean NRS score of 7.2 ± 2.1 in the USNB group and 6.9 ± 1.5 in the standard care group (*p* = 0.6). The distribution of triage acuity was also similar, with nearly all patients classified as emergent in both groups (100% vs. 97.7%, *p* = 1.0). Arrival via emergency medical technicians (EMT) occurred in one-third of the USNB group and 38.1% of the standard care group (*p* = 1.0). Across comorbidities—including diabetes mellitus (33.3% vs. 21.2%, *p* = 0.5), hypertension (25.0% vs. 18.8%, *p* = 0.7), chronic kidney disease (16.7% vs. 11.8%, *p* = 0.6), and chronic liver disease (16.7% vs. 8.2%, *p* = 0.3)—no statistically significant differences were observed. The prevalence of prior surgical history and malignancy was similarly low. However, the USNB group exhibited a significantly higher prevalence of chronic pain (16.7% vs. 0%, *p* = 0.01), representing the only baseline variable with a statistically significant between-group difference. Aside from chronic pain, no other demographic or clinical variable differed meaningfully across groups.

### 3.2. Comparison of Pain Scores Between the USNB and Standard Care Groups

**Figure 2:**
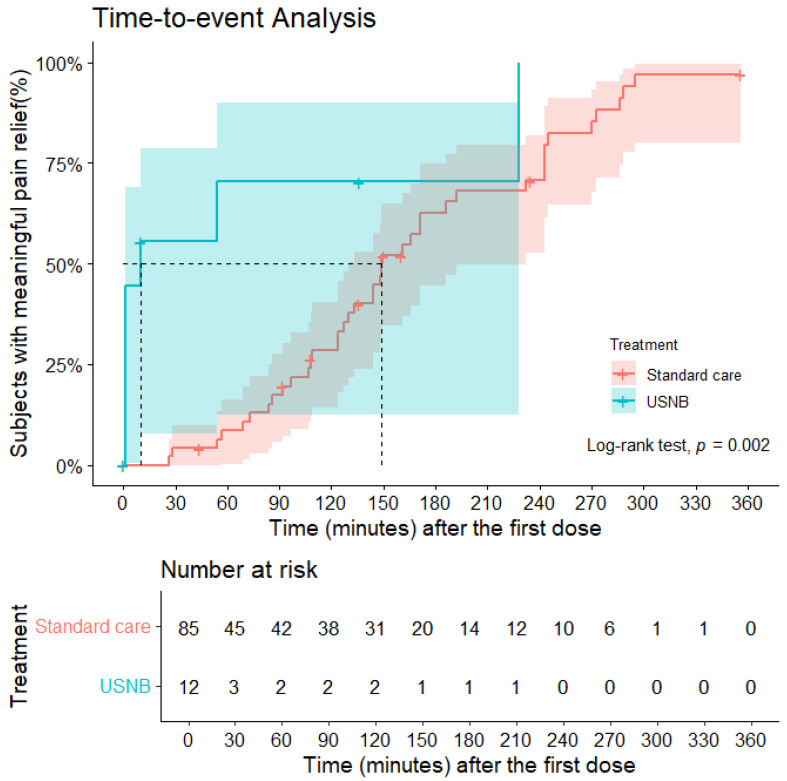
Kaplan–Meier plots for time to meaningful pain relief. Censoring (marked as “+”) occurred if a subject withdrew, received rescue analgesics, or did not experience meaningful pain relief during the entire observation period. Lines represent the proportion of patients achieving the outcome, and shaded areas represent 95% confidence intervals. Dashed lines indicate the timepoint at which 50% of patients in each group achieved meaningful pain relief. USNB: ultrasound-guided nerve block.

In the primary outcome analysis, USNB demonstrated substantially faster and more pronounced pain relief compared with standard care. The time-to-event analysis showed a clear early separation between the two groups (Figure 2). In the USNB group, 7 of 12 patients achieved meaningful pain relief during the observation window—defined as a pain intensity reduction of ≥4—with a median time of 10 min following the nerve block intervention. In contrast, in the standard care group, 39 of 85 patients experienced meaningful pain relief, with a median time of 149 min. Notably, in the USNB group, all observed event times for meaningful pain relief occurred within 240 min post-intervention (Figure 2). Patients receiving USNB achieved meaningful pain relief markedly earlier, with nearly half of the cohort reaching PID ≥ 4 within the first 10 min, whereas patients in the standard care group exhibited a gradual, delayed pain reduction over the 360-min follow-up period. The USNB curve demonstrated a steep early rise, reflecting rapid event accumulation, while the standard care curve showed a slow and progressive ascent. A log-rank test confirmed a statistically significant difference between the two survival curves (*p* = 0.002), indicating that USNB was associated with a markedly shorter time to clinically meaningful pain reduction. The Cox proportional hazards model demonstrated that patients receiving USNB achieved meaningful pain relief at a rate 3.41 times faster than those in the standard care group (HR = 3.41; 95% CI, 1.47–7.90; *p* = 0.004).

**Figure 3:**
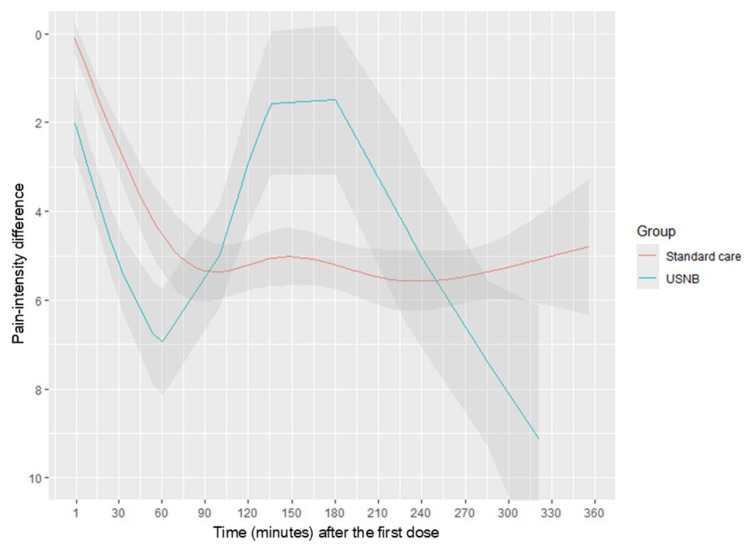
Pain score trajectory over time (minutes) between USNB group and standard group. Line represented the mean of PID and area as 95% confidence interval. PID: pain-intensity difference, USNB: ultrasound-guided nerve block.

To compare the evolution of PID values between groups across all assessment time points, we constructed a regression model to characterize the temporal trajectory of pain reduction. Non-linear associations between time and PID were examined using a restricted cubic spline with five knots. This time-course analysis showed that the USNB group exhibited a significantly greater and more rapid decline in pain scores throughout the 360-min observation window (*p* < 0.001) (Figure 3).

### 3.3. Secondary Outcomes

No significant differences were observed between the USNB and standard care groups regarding rescue opioid consumption, ED length of stay, or hospital length of stay (Table 2). The mean MME requirement was 3.88 ± 0.12 mg in the USNB group versus 7.66 ± 1.20 mg in the standard care group (mean difference −3.79 ± 4.96 mg, *p* = 0.45). A trend toward reduced opioid use was observed in the USNB group; however, this did not reach statistical significance. The length of ED stay did not differ significantly, with patients in the USNB group remaining for 5.47 ± 1.36 h versus 5.98 ± 0.92 h in the standard care group (mean difference −0.51 ± 2.51 h, *p* = 0.83). The hospital length of stay also showed no meaningful between-group difference, averaging 4.00 ± 0.82 days in the USNB cohort and 7.78 ± 0.93 days in the standard care group (mean difference −3.78 ± 2.32 days, *p* = 0.12). Overall, these findings indicate that USNB did not significantly reduce healthcare utilization in this cohort. No complications, such as vascular puncture, nerve injury, pneumothorax, hemidiaphragmatic paresis or local anesthetic systemic toxicity, related to the USNB procedure were observed.

## 4. Discussion

This study demonstrates that a single-shot USNB is an effective analgesic strategy for patients with acute clavicle fractures in the ED. USNB was found to provide significantly faster and more substantial pain relief in comparison with standard care with systemic analgesics. Patients receiving USNB achieved meaningful pain relief (PID ≥ 4) 3.41 times faster than those receiving standard care treatment, with a median time to relief of under 10 min. These findings are consistent with the broader, albeit limited, emergency medicine literature on this topic, in which reduced pain scores following RA for clavicle fractures has been consistently reported [11,18,19]. According to current evidence, this is the first study supporting the integration of USNB into ED protocols for rapid and effective pain management in patients with clavicle fractures.

The efficacy of the nerve block performed in our study is best understood in the context of the clavicle’s complex neuroanatomy [10,11,26]. Our USNB targets the superior and middle trunks of the brachial plexus (C5–C7) [11]. This approach is highly effective for bone and muscle pain, as it anesthetizes key nerves, such as the nerve to the subclavius, which are primary sources of deep, severe pain in clavicle fracture [10,11,13]. The priority in the ED is to control the severe pain originating from the bone and surrounding tissues, which our chosen technique effectively accomplishes. Our choice of lidocaine as the local anesthetic was strategic for the ED environment [7]. Compared with longer-acting agents like bupivacaine or ropivacaine, lidocaine has a more rapid onset of action, typically providing analgesia within approximately 3 min [7,23]. This speed is paramount in an acute setting to rapidly alleviate patient suffering [7]. The relatively shorter duration of action (approximately 4 h) is also advantageous, as it enables a more timely neurological and orthopedic assessment after the initial pain crisis has been managed, without the confounding effects of a prolonged motor or sensory block [7,23]. Furthermore, the use of ultrasound guidance enables precise perineural injection, which has been shown to reduce the minimum effective anesthetic concentration required, thereby enhancing the safety profile by minimizing the total dose of the drug [14,16,27].

Overall pain trajectories (Figure 3) differed significantly between USNB and standard care groups (*p* < 0.001, likelihood ratio test), with USNB demonstrating significantly superior analgesia within 120 min. However, the biphasic trajectory pattern observed in the USNB group warrants explanation. Two factors likely contributed: First, with only 12 patients in the USNB group, the restricted cubic spline model became sensitive to individual outliers, particularly pain score increases observed in several patients around 150 min. Second, our USNB protocol employed 1% lidocaine, which has a shorter duration of action compared with the standard 2% concentration, as demonstrated in previous studies [28].

Our findings demonstrate the safety and feasibility of EP-performed USNB. No procedure-related complications were observed, which aligns with a growing body of literature establishing ultrasound-guided RA as a safe technique with a lower risk of complications, such as inadvertent vascular puncture, in comparison with landmark-based methods [7,10,14,16]. The successful administration by trained EPs supports the expansion of this skill set within emergency medicine, which could improve patient flow and satisfaction by providing definitive pain management without requiring an immediate anesthesiology consult [9,11,14,16]. Importantly, and consistent with similar studies on hip fractures, the use of USNB did not prolong the ED length of stay, demonstrating that this intervention can be incorporated efficiently into existing workflows without causing delays [7,14].

A key advantage and motivation for using RA is its potential to reduce opioid consumption, a critical goal amid the ongoing opioid crisis [7,8,9,11,15,16]. Although we did not demonstrate a significant difference in rescue opioid use, the profound and rapid analgesia provided by USNB suggests a strong opioid-sparing potential [16,17]. Research has shown that an initial opioid prescription in the ED is associated with a significantly increased risk of long-term opioid use, particularly in older patients [8]. Furthermore, opioid use in this demographic is linked to higher rates of falls, fractures, and mortality [8,29,30]. Thus, interventions such as USNB, which align with an alternatives to opioids (ALTO)-first approach, are crucial. Studies investigating ALTO protocols have demonstrated that these methods can significantly decrease the administration of intravenous opioids without compromising patient satisfaction [9]. Despite the lack of statistically significant opioid reduction, the superior pain control achieved with USNB represents an important advancement in safer, multimodal pain management strategies that reduce reliance on opioid therapy [14,15,16].

Although our study focused on the interscalene block, some studies have indicated a combined approach using both an interscalene block and superficial cervical plexus block to achieve complete anesthesia of the overlying skin (dermatomes C3–C4) [10,11]. In addition, it is essential to note the emergence of alternative techniques, such as the clavipectoral plane block (CPB) [10,11,19]. The CPB is a more distal and superficial block that targets terminal nerve endings in the fascial plane around the clavicle, potentially offering a safer profile by avoiding proximity to the phrenic nerve and major vessels in the neck [10,11,19]. The development of such alternatives highlights the dynamic nature of regional anesthesia and presents future avenues for research in comparing the efficacy and safety of different blocks for this indication [10,11,13,19].

This study has several limitations. First, its retrospective, single-center, observational nature may introduce the potential for bias. Selection bias is a key concern, as the nonrandomized allocation of patients to the USNB or standard care group could have been influenced by unmeasured factors; for example, EPs may have been more inclined to perform a block on patients with higher initial pain scores or fewer comorbidities, a trend suggested in our baseline data. Although we used matching to adjust for observable differences, residual confounding may persist. Second, information bias could result from inconsistencies in the electronic health record, such as variable timing and documentation of pain scores. Third, the study involved a limited sample size. Post hoc power analysis based on our observed primary outcome event rates (60% achieving meaningful pain relief in USNB group vs. 50% in standard care) suggested that with alpha = 0.05, power = 0.80, and our 1:10 matching design, minimum required sample sizes were 5 intervention and 44 control patients [31]. Our actual sample (12 USNB patients and 120 matched controls) exceeded these minimum requirements. However, with only 12 patients receiving USNB, effect estimates remain imprecise, as reflected in the wide confidence interval for the primary outcome. Additionally, the small sample limited our ability to detect rare adverse events and made trajectory modeling sensitive to individual outliers. These preliminary findings require validation through larger prospective randomized trials to establish robust evidence, refine effect estimates, and inform clinical practice guidelines. Forth, because our study evaluated NRS scores only within the first 6 h, pain assessment beyond this time frame remains unknown and warrants further investigation. The treatments administered after 6 h were not examined in the present analysis, highlighting a potential area for future research. Finally, as this study was conducted at a single academic trauma center, the practice patterns and patient population may not be representative of other settings, such as community hospitals or different healthcare systems. Thus, while our results are promising, they should be interpreted with caution.

## 5. Conclusions

In this single-center cohort study of patients with traumatic clavicle fractures, a single-shot, lidocaine-based USNB provided more rapid and sustained pain relief than standard analgesic care in the ED. This intervention significantly reduced the time to meaningful pain control without increasing the ED length of stay or causing complications. These findings support the use of USNB as a safe, effective, and efficient non-opioid alternative for the acute pain management of clavicle fracture patients in the emergency setting. Further large, randomized controlled trials are warranted to confirm these observations and to further delineate the impact of USNB application on opioid consumption and other patient-centered outcomes.

## Figures and Tables

**Table 1:** Characteristics of patients who did and did not receive USNB.

Variables	USNB (n = 12)	Standard Care (n = 85)	*p*-Value
Mean (SD) age (years)	60.9 (14.7)	59.9 (13.9)	0.8
Woman, % (n)	50.0 (6)	45.9 (39)	0.8
Mean (SD) BMI	22.7 (3.4)	23.9 (4.6)	0.4
Mean (SD) of initial pain score	7.2 (2.1)	6.9 (1.5)	0.6
Triage *, % (n)			1.0
Emergent	100.0 (12)	97.7 (83)	
Urgent	0.0 (0)	2.6 (2)	
Arrival (sent by EMT), % (n)	33.3 (4)	38.1 (32)	1.0
Comorbidity			
DM, % (n)	33.3 (4)	21.2 (18)	0.5
HTN, % (n)	25.0 (3)	18.8 (16)	0.7
CAD, % (n)	0.0 (0)	0.0 (0)	1.0
CKD, % (n)	16.7 (2)	11.8 (10)	0.6
CLD, % (n)	16.7 (2)	8.2 (7)	0.3
Surgical History, % (n)	0.0 (0)	0.0 (0)	1.0
Chronic Pain, % (n)	16.7 (2)	0.0 (0)	0.01
Malignancy, % (n)	0.00 (0)	2.4 (2)	1.0

* Triage is known as Australasian Triage Scale, with 1 and 2 denoting emergent and 3–5 denoting urgent. Abbreviations: BMI = Body Mass Index; CAD = Coronary Artery Disease; CKD = Chronic Kidney Disease; CLD = Chronic Liver Disease; DM = Diabetes Mellitus; EMT = Emergency Medical Technician; HTN = Hypertension; SD = Standard Deviation; USNB = ultrasound-guided nerve block.

**Table 2:** Outcomes in Patients With and Without Ultrasound-guided Nerve Block for Clavicle Fracture.

		MMEs (mg)			Length of ED Stay (Hours)			Length of Hospital Stay (Days)		
	No	Mean	Difference	*p* Value	Mean	Difference	*p* Value	Mean	Difference	*p* Value
USNB	12	3.88 ± 0.12	−3.79 ± 4.96	0.45	5.47 ± 1.36	−0.51 ± 2.51	0.83	4.00 ± 0.82	−3.78 ± 2.32	0.12
Standard care	85	7.66 ± 1.20	reference		5.98 ± 0.92	reference		7.78 ± 0.93	reference	

The data were demonstrated after 1:10 matching. Unless otherwise specified, data are presented as mean (SE). ED, Emergency department; MME, morphine milligram equivalents; SE, standard error; USNB, ultrasound-guided nerve block.

## Data Availability

The data supporting the findings of this study are available from the corresponding author upon reasonable request for research purposes only. Access to the dataset requires submission of appropriate documentation demonstrating institutional approval or ethical clearance. Interested researchers may contact the corresponding author to initiate the request process.
